# Perspectives of Pulmonologists on the 2009-2010 H1N1 Vaccination Effort

**DOI:** 10.1155/2012/306207

**Published:** 2012-01-04

**Authors:** Sarah J. Clark, Anne E. Cowan, Pascale M. Wortley

**Affiliations:** ^1^Child Health Evaluation and Research (CHEAR) Unit, University of Michigan, 300 N Ingalls, Room 6E06, Ann Arbor, MI 48109-5456, USA; ^2^Immunization Services Division, NCIRD, Centers for Disease Control and Prevention, Atlanta, GA 30333, USA

## Abstract

Persons with high-risk conditions such as asthma were a target group for H1N1 vaccine recommendations. We conducted a mailed survey of a national sample of pulmonologists to understand their participation in the 2009-2010 H1N1 vaccine campaign. The response rate was 59%. The majority of pulmonologists strongly recommended H1N1 vaccine for children (73%) and adults aged 25–64 years (51%). Only 60% of respondents administered H1N1 vaccine in their practice compared to 87% who offered seasonal influenza vaccine. Other than vaccine supply, respondents who provided H1N1 vaccine reported few logistical problems. Two-thirds of respondents would be very likely to vaccinate during a future influenza pandemic; this rate was higher among those who provided H1N1 vaccine and/or seasonal flu vaccine. In total, the H1N1 vaccine-related experiences of pulmonologists seemed to be positive. However, additional efforts are needed to increase participation in future pandemic vaccination campaigns.

## 1. Introduction

In June 2009, the World Health Organization declared a worldwide pandemic of H1N1 influenza [1], and, in September 2009, a novel H1N1 influenza vaccine was licensed for use in the United States [2]. As with seasonal influenza, persons with asthma and other chronic respiratory conditions were considered at high risk for H1N1 influenza and its complications [3].

Early reports indicated high rates of asthma among those hospitalized and a younger age profile among those hospitalized than what is common for seasonal influenza [4–6]. These data underscored the importance of vaccinating high-risk children and nonelderly adults, as well as the need for effective strategies to reach these populations. In this context, pulmonologists represented a provider group that could vaccinate children and adults with asthma and other high-risk respiratory conditions.

Unlike a typical influenza season, in which physicians who offer flu vaccine purchase it in the private market, the federal government purchased H1N1 vaccine and gave state and local public health officials the responsibility for coordinating the H1N1 vaccination campaign in their jurisdiction. These responsibilities included enrollment of providers as H1N1 vaccinators and distribution of H1N1 to enrolled providers. The extent to which state and local public health officials were able to involve subspecialists in the H1N1 vaccination campaign is unknown.

The goal of this study was to describe the experiences of pulmonologists related to the 2009-2010 H1N1 vaccination campaign to help inform future influenza vaccination campaigns.

## 2. Methods

### 2.1. Sample

Through a contracted vendor, we drew a national random sample of 1,998 pulmonologists from the American Medical Association (AMA) Physician Masterfile. The AMA Physician Masterfile is a comprehensive database of physicians licensed to practice in the United States, and includes both AMA members and nonmembers. Our sampling frame included all allopathic and osteopathic physicians in office- or hospital-based, direct patient care, with a self-reported primary specialty of pulmonary disease. Excluded were physicians ≥70 years, in residency training or employed at federal medical facilities.

### 2.2. Survey Design

The 4-page, 18-item survey instrument incorporated factors known to be related to influenza vaccination in general (e.g., strength of physician recommendation for vaccination [4–10], safety concerns about influenza vaccine [4, 5, 8]), as well as practical and logistical issues specific to the H1N1 vaccine campaign. Specific questions included the following:

- typical recommendation regarding H1N1 vaccination for patients by age group (*strongly recommended, recommended, neutral, recommended against*) and whether recommended H1N1 vaccine for patients during inpatient stays,
- own and perceived patient level of concern with safety of H1N1 vaccine relative to that of seasonal flu vaccine (*much less, somewhat less, about the same, somewhat greater, much greater*),
- level of participation in the 2009-2010 H1N1 vaccine campaign (*agreed to provide H1N1 vaccine and received vaccine*, *agreed to provide H1N1 vaccine but did not receive vaccine, did not agree to provide H1N1 vaccine, and did not know about opportunities to provide H1N1 vaccine*),
- among H1N1 vaccinators, experiences with administering H1N1 vaccine, including month vaccine first available for patients, whether prioritized among adult patients when supply was limited and, if so, to which subgroups, and extent to which certain practical and logistical issues related to H1N1 vaccination were problematic (*not a problem, minor problem, major problem*),
- whether practice offered seasonal flu vaccine in 2009-2010 and planned to offer flu vaccine for the 2010-2011 influenza season,
- likelihood of practice providing vaccine in the event of a future influenza pandemic (*very likely, somewhat likely, neutral, somewhat unlikely, very unlikely*), and
- practice characteristics, including practice ownership/affiliation and whether provide inpatient care.

The University of Michigan Medical School Institutional Review Board approved this study.

### 2.3. Survey Administration

The initial survey mailing was sent in June 2010 and included a $5 cash incentive. Two additional mailings to nonrespondents occurred at approximately 4-week intervals.

### 2.4. Data Analysis

We generated univariate frequencies for each variable and then performed chi-square analyses to examine associations between key survey variables (e.g., H1N1 vaccine recommendation, H1N1 vaccinator status, likelihood of participating in future influenza pandemic) and practice and personal demographic characteristics. A two-tailed *α*-level of 0.05 was the threshold for statistical significance. All analyses were conducted using SAS version 9.2 (SAS Institute, Cary, NC, USA).

## 3. Results

### 3.1. Response Rate

Of the 1,998 pulmonologists in the mailing sample, 107 were excluded because mailing materials were returned as undeliverable. Completed surveys were returned by 1,114 pulmonologists, yielding a response rate of 59%. Of these, 169 were ineligible because they do not provide any direct patient care in an outpatient setting. Thus, the final analytic sample included 945 respondents. Practice characteristics of respondents are presented in Table 1.

### 3.2. H1N1 Vaccine Recommendation

Respondents' strength of recommendation for H1N1 vaccine varied by patient age group (Table 2), with a strong recommendation most common for pediatric patients. There were no consistent or notable significant differences in the strength of H1N1 vaccine recommendation by practice characteristics for any of the three patient age groups.

### 3.3. H1N1 Vaccinator Status

As shown in Table 3, the majority (60%) of respondents received H1N1 vaccine to administer to patients (i.e., were H1N1 vaccinators), while a small proportion agreed to participate but never received vaccine. Nearly one-third of respondents did not agree to participate or had not heard of opportunities to participate in the H1N1 vaccine campaign. H1N1 vaccinator status was associated with practice setting; all respondents in public clinics and three-quarters of those based in hospitals were H1N1 vaccinators. However, in private practice, the most common practice setting, just over half of respondents were H1N1 vaccinators. Pulmonologists whose practices offered seasonal flu vaccine in 2009-2010 were much more likely to be H1N1 vaccinators; less than 10% of those who did not offer seasonal flu vaccine were H1N1 vaccinators.

### 3.4. Vaccine Safety Concerns

Eleven percent of respondents reported that they had greater concerns about the safety of H1N1 vaccine than seasonal influenza vaccine, 59% had “about the same” level of concern, and 30% had less concern about the safety of H1N1 vaccine. Respondents who had a greater concern about H1N1 vaccine safety were less likely to strongly recommend H1N1 vaccine than those whose level of concern was similar or lower; this finding was consistent for all three patient age groups (*P* = .018 for pediatric patients, and *P* < .001 for both adults 25–64 years and seniors). In addition, respondents who reported safety concerns were less likely to be H1N1 vaccinators compared to those with a similar or lower level of concern (36% versus 63%, *P* < .001).

With regard to patients' safety concerns, 52% of respondents perceived that patients were more concerned with the safety of H1N1 vaccine relative to seasonal influenza vaccine. Another 33% felt that their patients' level of concern was “about the same,” while 15% perceived patient concerns to be less for H1N1 vaccine than seasonal flu vaccine. Perceived patient concern was not associated with physicians' self-reported strength of recommendation for H1N1 vaccine or H1N1 vaccinator status.

### 3.5. Experiences of H1N1 Vaccinators

Among H1N1 vaccinators, the first month in which respondents reported having vaccine available for patients was 20% October, 34% November, 20% December, and 7% January or later; 19% were unsure of the timing of vaccine availability. Most H1N1 vaccinators offered vaccine at scheduled visits (74%) or on a walk-in basis (62%), with just under half (45%) offering both options.

Based on the practical and logistical issues with H1N1 vaccination listed in Table 4, vaccine supply was the main challenge for H1N1 vaccinators. Across all of the issues listed in Table 4, 39% of respondents rated one of these issues as a major problem, 13% said two of these issues were major problems, 7% rated three or more as major problems, and 41% of respondents reported no major problems.

### 3.6. Future Participation in Influenza Vaccination

Most respondents (86%) reported that their practice planned to offer seasonal flu vaccine for the 2010-2011 influenza season, which was similar to the proportion offering seasonal flu vaccine for the 2009-2010 season (87%). A smaller proportion (65%) said their practice would be “very likely” to provide vaccine in the event of a future influenza pandemic, with another 13% indicating their practice would be “somewhat likely” to participate. An additional 8% were neutral, 5% said “somewhat unlikely,” and 9% said “very unlikely.”

Being “very likely” to provide vaccine in a future influenza pandemic was more common among those who offered versus did not offer seasonal flu vaccine during the 2009-2010 influenza season (72% versus 19%, *P* < .001) and among H1N1 vaccinators versus nonvaccinators (84% versus 34%, *P* < .001). Among H1N1 vaccinators, being “very likely” to participate in a future influenza pandemic was less common among those who rated two or more of the issues in Table 4 as major problems compared to those who said none or one of the issues was a major problem (64% versus 86%, *P* = .002).

## 4. Discussion

Summary data on hospitalizations and deaths resulting from the 2009-2010 H1N1 influenza [11, 12] confirmed initial reports [4–6] that persons with asthma and other chronic conditions were at higher risk for morbidity and mortality and, therefore, an important target for H1N1 vaccination. One strategy for reaching these populations was to include subspecialists, such as pulmonologists, in the 2009-2010 H1N1 vaccination campaign.

Assessing physician participation in public health campaigns is a critical aspect of understanding the extent to which a shared public health-medical delivery system is feasible for future pandemic-type events. A positive finding from this study is that a majority of pulmonologists surveyed participated in the 2009-2010 H1N1 vaccination campaign as H1N1 vaccinators. The importance of including physicians that can reach high-risk populations is supported by recent data showing that persons at high risk are more likely than those not at high risk to receive flu vaccine at a doctor's office versus other locations [13].

Of concern, however, is that participation was lowest in private pulmonology practices (respondents' most common practice setting) relative to hospital-based and public sector practices. This highlights a challenge for state and local public health officials in recruiting physicians to assist with pandemic vaccination campaigns. While these officials may be able to build upon existing relationships with hospitals to reach hospital-based providers, recruiting private practices is likely more time and resource intensive, as each practice must be contacted individually. In addition, from the physicians' perspective, smaller independent practices may be less likely to want, or to have the capacity, to take on an additional vaccine.

Participation in the H1N1 vaccination campaign among those who did not provide seasonal flu vaccine in 2009-2010 was much lower, and approximately one-third of these respondents said they did not know about opportunities to provide H1N1 vaccine, suggesting that it is particularly difficult to reach those who are not already “plugged in” to the immunization delivery system. For this reason, public health officials may find it easier to recruit primary care physicians for pandemic vaccination efforts. However, including subspecialists is important for reaching persons at high risk. For example, although the majority of ambulatory care visits for asthma and other chronic pulmonary conditions are to primary care offices, about one-third of these visits are to medical subspecialty offices [14]. Focusing solely on primary care physicians would likely miss a subset of this high-risk population.

Overall, a high proportion of pulmonologists recommended H1N1 vaccine to their patients. Given that virtually all of their patients less than 65 years of age would be classified as high risk for H1N1 vaccine, it is unclear why the proportion who “strongly recommended” vaccination was much higher for patients 18 years and younger compared to those aged 25–64 years. Because all children were an initial target group for vaccination, not just the high-risk subgroup, it may have been easier for physicians to emphasize vaccination for this patient group. It is also possible that state or local public health or medical community messages may have focused more attention on vaccinating children.

The proportion of pulmonologists who strongly recommended H1N1 vaccine to adults aged 25–64 years was only slightly higher than the proportion for adults aged 65 years and older, despite the fact that the latter population was not an initial target group for H1N1 vaccination. Expansion beyond the initial target groups was recommended first for all adults aged 25–64 years and then, once demand for the younger age groups was met, to those aged 65 years and over, regardless of high-risk status [3]. It is possible that some pulmonologists may have been unaware of the differential recommendations for these populations, or that, by the time vaccine was available in their practice setting, the target group recommendations were no longer needed. It is also possible that some pulmonologists disagreed with the recommendations, feeling that the presence of a high-risk pulmonary condition preempted an age-based determination.

While the proportion of respondents having greater concerns with the safety of H1N1 vaccine relative to seasonal flu vaccine was not large, those with greater concerns were much less likely to strongly recommend H1N1 vaccine to their patients and participate as H1N1 vaccinators. Future campaigns should stress the importance of proactive and widespread messages directed toward physicians regarding vaccine safety.

By far the biggest challenge faced by H1N1 vaccinators was the limited supply of vaccine. Although the majority of respondents reported receiving vaccine by November, the peak of disease, and likely of patient demand, occurred prior to widespread vaccine availability. This was a significant and broadly experienced challenge during the H1N1 vaccination campaign [15] and also varied by state or local area. Otherwise, few issues caused major problems for pulmonologists providing H1N1 vaccine, and many reported experiencing no major problems.

A majority of respondents felt their practice would be “very likely” to provide vaccine in the event of a future influenza pandemic. Respondents who were H1N1 vaccinators were much more likely to express willingness to participate in the event of a future pandemic, as were those who provided seasonal flu vaccine in 2009-2010. Although the proportion very likely to participate in a future influenza pandemic was similar to the proportion that was H1N1 vaccinators, it is markedly lower than the proportion providing seasonal flu vaccine, implying that outpatient pulmonologists do not see themselves as essential to a pandemic response. Public health and emergency preparedness officials may want to work toward including subspecialty physicians, such as pulmonologists, in short-term planning efforts, in order to accomplish longer-term objective of creating a broad base of vaccinators for pandemic response. In addition, pulmonologists interested in vaccinating can be proactive in reaching out to public health officials during a pandemic. Strategies for encouraging pulmonologists' participation in pandemic vaccination should continue to be explored.

## 5. Limitations

As with any survey study, there are limitations of response bias; however, the response rates obtained in this study were quite favorable, compared to other physician surveys. Also, given the sample size, we were unable to make statistically viable state-by-state comparisons of the results. The major limitation with this study is that our pulmonologist sample is not generalizable to the full universe of pulmonologists. We included physicians in our sample whose self-reported primary specialty is pulmonary disease but did not include the other potentially applicable primary specialty of pulmonary critical care medicine. To be eligible for our study, respondents needed to provide patient care in an outpatient setting, and we felt that a high proportion of physicians reporting pulmonary critical care medicine as their primary specialty would not provide outpatient care. As it turns out, respondents reporting a specialty of pulmonary disease are skewed to older, male physicians; the sample included no one under the age of 40 years. In exploring this issue further, we found that recent research has shown that pulmonology alone is not a popular choice among internal medicine residents and that pulmonology with critical care is more likely to be chosen than either pulmonology or critical care alone [16]. We went back to the contracted vendor and requested year of birth frequency counts from the AMA Masterfile, using our initial sample specifications, for all physicians reporting a primary specialty of either pulmonary critical care medicine or pulmonary disease. Comparing year of birth data across the two groups confirmed that younger physicians are reporting pulmonary critical care medicine as their primary specialty to the exclusion of pulmonary disease. Given this limitation, our results likely under-report the experiences of hospital-based pulmonologists and likely provide a conservative estimate of pulmonologists' participation in the H1N1 vaccination campaign.

## 6. Conclusion

Federal, state, and local public health officials should be encouraged to include pulmonologists in future pandemic influenza planning and vaccination efforts as an effective strategy for reaching persons with asthma and other chronic respiratory conditions. The experiences of a subset of pulmonologists in the H1N1 vaccine campaign were generally positive, with the majority recommending the vaccine to their patients and serving as H1N1 vaccinators. However, there is much room for improvement in terms of participation levels, and the challenges related to participation by private practices must be further explored.

## Figures and Tables

**Table 1:** Respondent characteristics (*N* = 945).

	**%**
Practice setting/affiliation	
Private independent practice/network, IPA	82%
Hospital, Health system, Univ. medical center	17%
Public clinic, Community health center, other	1%

Patient mix	
Pediatric and adult patients	63%
Adult patients only	37%

Provide any inpatient care	
Yes	91%
No	9%

Offered seasonal flu vaccine during the 2009-2010 influenza season	
Yes	87%
No/Unsure	13%

**Table 2:** Pulmonologists' typical H1N1 vaccine recommendation by patient age group (*N* = 945)*.

For patients	Strongly recommended	Recommended	Neutral	Recommended against
≤18 Years	73%	18%	7%	2%
25–64 Years	51%	39%	9%	1%
≥65 Years	43%	33%	18%	6%

*Results for each age group exclude respondents who do not provide care for patients of that age.

**Table 3:** H1N1 vaccinator status of pulmonologists and association with practice characteristics.

Variable	Agreed to participate, received vaccine	Agreed to participate, did not receive vaccine	Did not agree to participate	Did not know about opportunities to participate
Overall (*N* = 945)	60%	9%	20%	11%

Practice setting/affiliation^∗†^				
Private independent practice/network (*N* = 760)	56%	10%	23%	11%
Hospital/health system (*N* = 165)	77%	5%	9%	9%
Public clinic/CHC/other (*N* = 6)	100%	0%	0%	0%

Offered seasonal flu vaccine for 2009-2010				
influenza season^∗†‡^				
Yes (*N* = 805)	68%	10%	14%	8%
No (*N* = 122)	9%	2%	59%	30%

*P < .001.

^†^CHC: community health center; *N* = 14 missing data.

^‡^Excludes “Unsure” category (*N* = 2); *N* = 16 missing data.

**Table 4:** Extent of problems experienced by pulmonologists providing H1N1 vaccine (*N* = 564).

	Not a problem	Minor problem	Major problem
Inadequate or inconsistent H1N1 vaccine supply	18%	37%	45%
Lack of patient acceptance (demand) for H1N1 vaccine	50%	42%	8%
Elderly patients frustrated with lower priority for vaccine	39%	48%	13%
Staff unfamiliar with different H1N1 vaccine products	78%	21%	1%
Storage/handling of H1N1 vaccine	83%	15%	2%
Reimbursement/billing for vaccine administration	70%	24%	6%
H1N1 reporting requirements	51%	39%	10%
Return/disposal of unused H1N1 vaccine	66%	27%	7%
